# Application of surface-enhanced resonance Raman scattering (SERS) to the study of organic functional materials: electronic structure and charge transfer properties of 9,10-bis((*E*)-2-(pyridin-4-yl)vinyl)anthracene†

**DOI:** 10.1039/c9ra01269a

**Published:** 2019-05-09

**Authors:** Juan Soto, Elizabeth Imbarack, Isabel López-Tocón, Santiago Sánchez-Cortés, Juan C. Otero, Patricio Leyton

**Affiliations:** Department of Physical Chemistry, Faculty of Science, Andalucía Tech, Unidad Asociada IEM-CSIC 29071-Málaga Spain soto@uma.es; Instituto de Química, Pontificia Universidad Católica de Valparaiso Valparaiso Chile; Instituto de Estructura de la Materia, IEM-CSIC Serrano 121 28006-Madrid Spain

## Abstract

The electron donor–acceptor properties of 9,10-bis((*E*)-2-(pyridin-4-yl)vinyl) anthracene (BP4VA) are studied by means of surface-enhanced Raman scattering (SERS) spectroscopy and vibronic theory of resonance Raman spectroscopy. The SERS spectra recorded in an electrochemical cell with a silver working electrode have been interpreted on the basis of resonance Raman vibronic theory assisted by DFT calculations. It is demonstrated that the adsorbate–metal interaction occurs through the nitrogen atom of the pyridyl moiety. Concerning the electron donor–acceptor properties of the adsorbate, it is shown that the charge transfer excited states of BP4VA are not optically active, in contrast, an internal transition to an excited state of BP4VA, which is localized in the anthracene framework, is strongly allowed. The charge transfer states will be populated by an ultrafast non-radiative process, that is, internal conversion. Thus, irradiation of BP4VA interacting with an appropriate surface creates an effective charge separation.

## Abstract 2

Surface enhanced Raman spectroscopy in conjunction with quantum chemistry is a valuable tool for characterization of organic functional materials.

## Graphical abstract

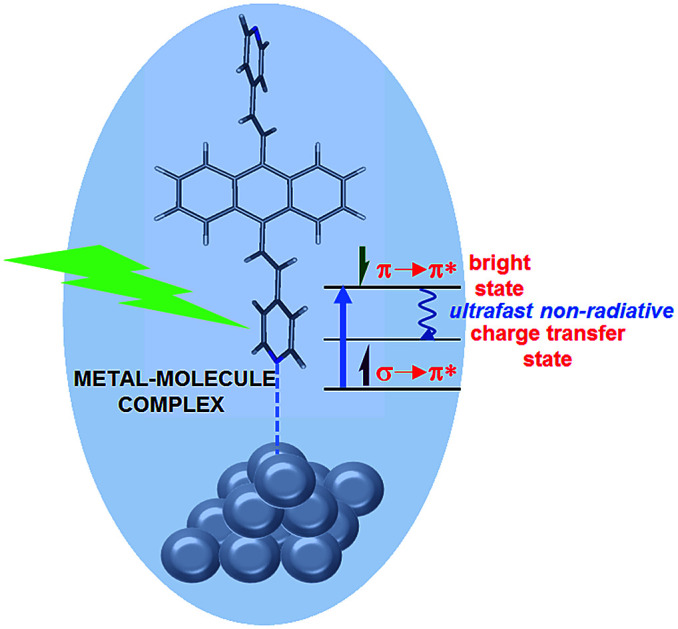

## Introduction

**Scheme 1:**
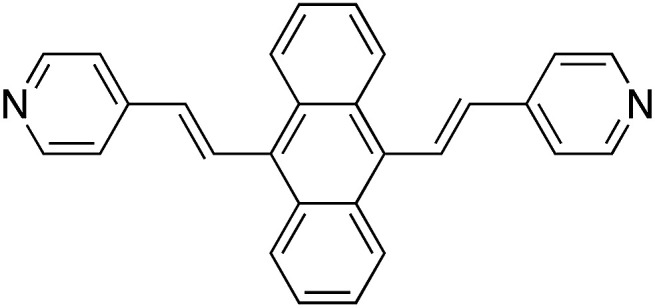
9,10-Bis((*E*)-2-(pyridin-4-yl)vinyl).

The synthesis of 9,10-bis((*E*)-2-(pyridin-4-yl)vinyl) anthracene (BP4VA, Scheme 1) was reported for the first time by Tian and coworkers in 2013.^1^ BP4VA is a material with interesting properties such as stimuli-responsive luminescence^1^ or semiconducting^2^ properties and is a potential candidate for several applications, for example, sensing, detection, display devices,^1^ organic solar cells^2,3^ or fluorescence live cell imaging^4,5^ to name a few. All of these properties and applications are controlled by the electronic structure and charge transport availability of the material under consideration. In this context, both surface- enhanced Raman scattering (SERS) and theory are valuable tools to explore such donor–acceptor electron features. Thus, the present work focuses on the analysis of the surface-enhanced Raman scattering (SERS) spectra^6,7^ of BP4VA recorded on an electrochemical cell under a linear variation of the working silver electrode potential in conjunction with calculations of theoretical spectra with programs developed and implemented by us.

Irradiation of a molecule in the proximity of a roughened or nanostructured noble metal surfaces origins the so-called surface-enhanced Raman scattering (SERS) effect, which was discovered by Fleischmann *et al.*^6,7^ The most remarkable characteristic of this phenomenon consists in a huge intensification of the weak Raman signal. Although a deep understanding of the phenomenon has not still been achieved, advances in the knowledge of SERS mechanisms and in the preparation of substrates has enabled the development of practical applications of this spectroscopic technique.^8–11^

The processes that are responsible for the signal enhancements in SERS are classified in two categories: (i) the electromagnetic (plasmonic/EM) or physical mechanism, which is always present in SERS, and (ii) the chemical mechanism (CM). Nowadays, it is widely accepted that localized surface plasmons (LSP) are responsible for EM mechanism. The EM mechanism explains the large (up to 10 magnitude orders)^5^ and uniform enhancement of the Raman spectrum, without modifying the relative intensities of the bands. For example, this is the case for the SERS spectra of pyridine registered on silver electrode at 0 and −0.25 V (*λ*_exc_ = 514.5 nm), where the relative intensities of the observed bands do not differ noticeably from those of the Raman spectra recorded in aqueous solution. In contrast, the chemical mechanism explains the selective intensification of specific bands of the Raman spectra.^5^

The chemical contribution can be divided in several types: (i) effects due to the adsorption process of the molecule in its electronic ground state; (ii) charge-transfer mechanism (SERS-CT), which involves an internal resonant electronic transition of the complex formed by the adsorbed molecule (A) and the metal (M), in either of the two directions, A ↔ M, molecule to metal or metal to molecule (A^−^–M^+^);^12–23^ (iii) surface-enhanced resonance Raman scattering (SERRS),^24,25^ when the laser wavelength matches the energy of an internal electronic transition of the adsorbed molecule; (iv) plasmon-like resonant mechanism (PL-SERS),^26^ a mechanism which was recently introduced by us and consists of a selective intensification of certain bands due to the coupling of internal resonant excitations of small metal clusters with specific normal modes of the adsorbate. It is important to note that, in essence, the SERS-CT contribution is part of the general SERRS process which involves the three mentioned different kinds of resonances (ii–iv); however, we introduce a distinction between them for the sake of clarity.

## Results and discussion

The main problem when analyzing a SERS is to know which mechanism or mechanisms are acting in each particular experiment. In this respect, the comparison between the relative intensities in the Raman and SERS spectra is the only way to detect which contribution is responsible for the changes in the Raman selection rules. Our group has proposed a theoretical methodology based on quantum chemical calculations able to predict the relative intensities of a SERS spectrum under applied electric potential. If a correlation is found between experimental and calculated behavior it is possible to confirm the participation of a type of resonant process in the spectrum.

### Normal Raman spectrum

**Fig. 1:**
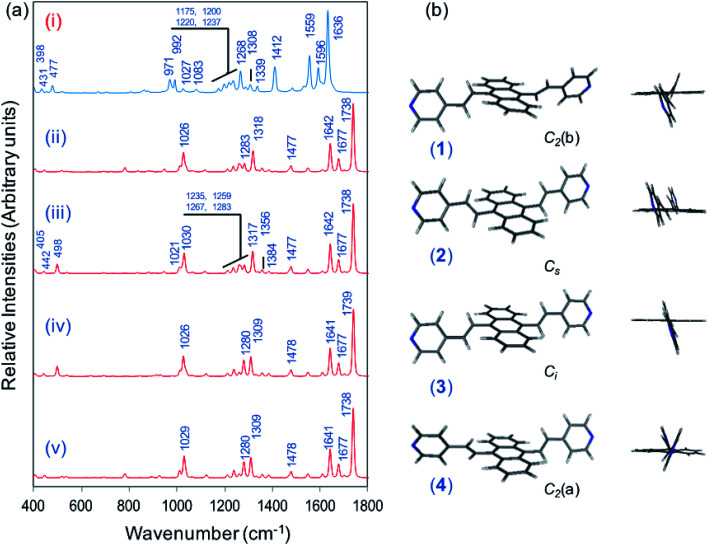
(a) Observed and calculated Raman spectra of BP4VA at *λ*_exc_ = 1064 nm: (i) experimental (solid sample); (ii) *C*_2_(b)-BP4VA; (iii) *C*_s_-BP4VA; (iv) *C*_i_-BP4VA; (v) *C*_2_(a)-BP4VA. Calculated spectra convoluted with a Voigt function (1 : 1) of HWHM = 5 cm^−1^. (b) Calculated CAM-B3LYP/def2-TZVPP conformers of BP4VA: (1) *C*_2_(b)-BP4VA; (2) *C*_s_-BP4VA; (3) *C*_i_-BP4VA; (4) *C*_2_(a)-BP4VA.

Fig. 1a displays the normal Raman (NR) spectrum of the solid sample of BP4VA (obtained at *λ*_exc_ = 1064 nm to avoid fluorescence emission) along with the calculated spectra of the four stable conformers optimized with the CAM-B3LYP/def2-TZVPP method. BP4VA is poorly soluble in water and it was not possible to record the aqueous solution Raman spectrum.

**Fig. 2:**
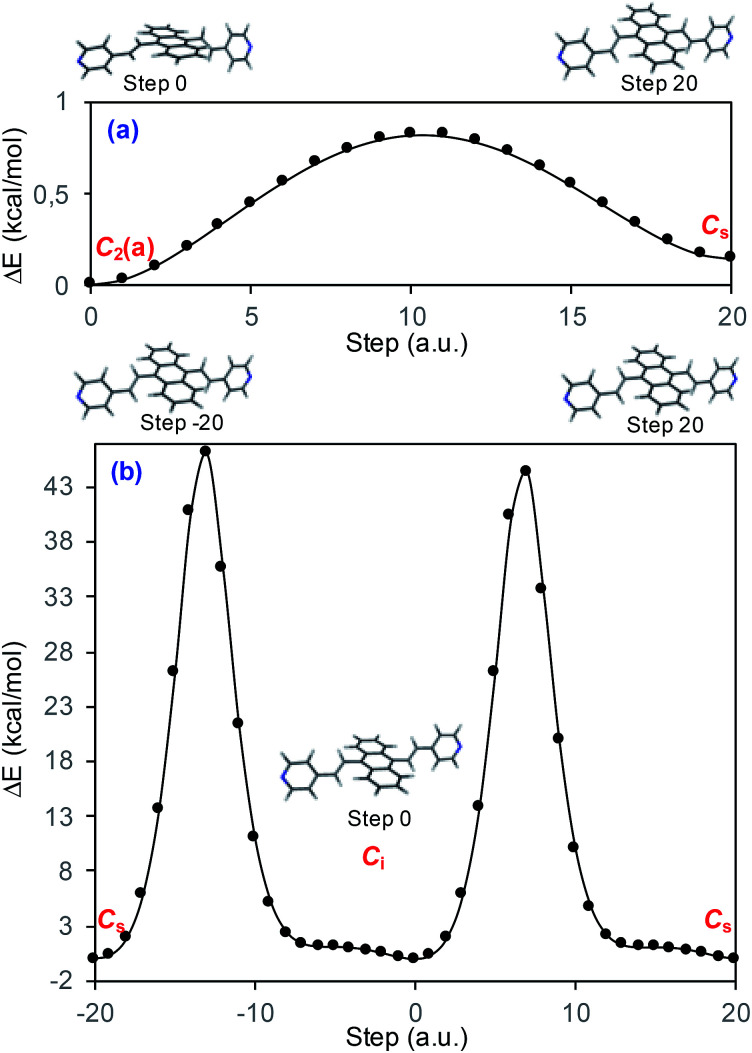
CAM-B3LYP/def2-TZVPP profiles of the potential energy surfaces for isomerization of (a) *C*_s_ to *C*_2_(a) and (b) *C*_s_ to *C*_i_.

Vibrational assignment of the bands of the spectra of BP4VA has been assisted by DFT (CAM-B3LYP/def2-TZVPP) calculations and with the help of the graphical representation of the normal modes (Fig. S2†) by means of the MacMolplt program.^27^ The calculations of the minimum energy geometries predict that the isolated molecule has four stable conformers almost isoenergetic with different molecular symmetries (Fig. 1b). The lowest energy conformer corresponds to the *C*_2_-symmetry geometry labeled as *C*_2_(a) [(4) in Fig. 1b]. Table S1† collects the vibrational analysis of the Raman active bands of both the experimental and calculated spectra. The calculated wavenumbers have not been scaled in Table S1.† The agreement between the experimental and calculated spectra for each one of the four conformers is quite satisfactory (Fig. 1a). In addition, it is found that the predicted spectra are almost independent of the excitation line included in the calculations (Fig. S1†). The four calculated spectra are almost coincident, however, the best agreement between the experimental and calculated spectra corresponds to the *C*_s_-conformer [(2) in Fig. 1b], which is only 0.1 kcal mol^−1^ above the most stable *C*_2_-isomer [(4) in Fig. 1b]. Therefore, if we accept that the starting conformer is the *C*_s_-isomer, it is interesting to explore the energy barriers for the interconversion from one isomer to the others. Thus, Fig. 2 represents the energy barrier heights for *C*_s_ ↔ *C*_2_(a) and *C*_s_ ↔ *C*_i_ isomerization, respectively, which have been calculated by means of the linear interpolation method performed in internal coordinates.^28–34^ The energy barrier for the *C*_s_ ↔ *C*_2_(a) reaction amounts to only 0.8 kcal mol^−1^ (Fig. 2a). In contrast, the *C*_s_ ↔ *C*_i_ isomerization displays a barrier energy height of 46 kcal mol^−1^ due to steric hindrance between the protons of the anthracene and vinylene groups (Fig. 2b). Therefore, while it is expected that the *C*_2_(a) and *C*_s_ isomers coexist in solution, the isomerization of the starting *C*_s_-conformer to *C*_i_ or *C*_2_(b) is hindered and it is unlikely to find them in solution.

### Calculated resonance Raman spectra of the isolated molecule

**Fig. 3:**
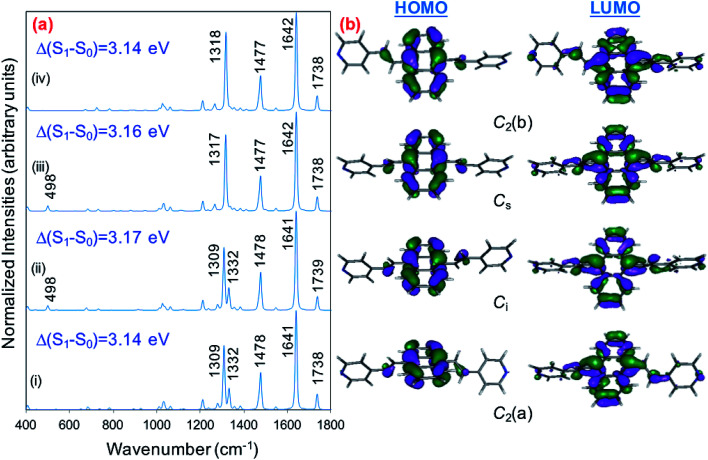
(a) CAM-B3LYP/def2-TZVPP resonance Raman spectra of BP4VA at *λ*_exc_ = 514.5 nm. (i) *C*_2_(a)-BP4VA; (ii) *C*_i_-BP4VA; (iii) *C*_s_-BP4VA; (iv) *C*_2_(b)-BP4VA. (b) Main orbitals involved in the π–π* transition (S_0_–S_1_) for the four conformers. Spectra convoluted with a Voigt function (1 : 1) of HWHM = 5 cm^−1^.

Prior to the analysis of the SERS spectra, we have studied the resonance Raman (RR) spectra of the isolated molecules, which are represented in Fig. 3a for the four conformers. The RR spectra have been calculated according to the multi-state version of the vibronic theory of Albrecht,^35–37^ (more details are given in the computational details section). Although we have included twenty electronic states in computing the spectra, it is found that the largest weight corresponds to the first excited state (S_1_) at *λ*_exc_ = 514.5 nm, which is described by a HOMO–LUMO (π → π*) transition (Fig. 3b). The calculated intensities of the bands of the RR spectra, given in Fig. 3, contrast with the NR bands of Fig. 1a (experimental and calculated). For example, the most intense bands in resonant conditions corresponds to 8a[1] and 1 modes of anthracene, where electronic excitation is localized. In contrast, the relative intensity of the *ν*(C=C) mode of vinylene decreases noticeably and 8a mode of pyridine is absent.

### SERS spectra on silver electrode

**Fig. 4:**
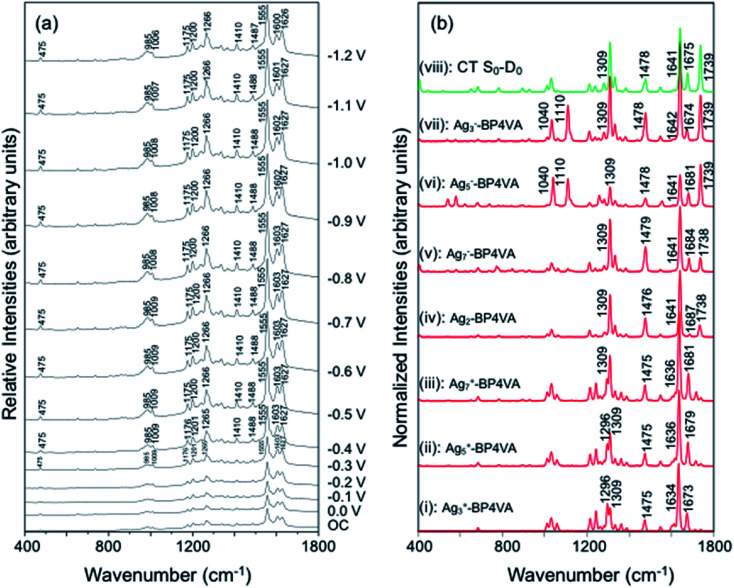
(a) SERS spectra at *λ*_exc_ = 514.5 nm of BP4VA/Na_2_SO_4_ (10^−4^ M/0.1 M) aqueous solution on silver at different electrode potentials (reference electrode Ag/AgCl/KCl sat.). (b) CAM-B3LYP/def2-TZVPP spectra of *C*_2_(a)-BP4VA–metal complexes (*λ*_exc_ = 514.5 nm); on top (green) S_0_–D_0_ charge transfer spectrum of the isolated molecule. Calculated spectra convoluted with a Voigt function (1 : 1) of HWHM = 5 cm^−1^.

The spectra of BP4VA (10^−4^ M) in aqueous solution registered in SERS conditions on silver electrode at different potentials (*λ*_exc_ = 514.5 nm) are shown in Fig. 4a. Comparison of the intensities of the normal Raman spectrum of the solid (Fig. 1a) with the spectra obtained on silver electrodes (Fig. 4) plus the fact that it is not observed Raman signals from the aqueous solution clearly indicate that we are observing a SERS phenomenon.

In addition, there is selective enhancement of particular bands of the SERS spectra with respect to the Raman spectrum of the solid.

Thus, in this section, we analyze the SERS spectra of BP4VA obtained at different electrode potentials (from 0 V up to −1.2 V and open circuit) in an electrochemical cell with a silver working electrode. The intensities of the spectra in Fig. 4a are shown as they are recorded, without any further mathematical treatment. Three features of these spectra must be remarked. First, it is observed that the absolute intensities of the spectra increase as the electrode potential becomes more negative. Second, the line shape of the intensified modes is not so sharp as in other SERS spectra containing pyridine derivatives.^14–17^ Third, the relative intensities of the bands depend moderately on the electrode potential; this is especially notable for the 8a mode of pyridyl moiety (∼1600 cm^−1^), which is found strongly sensitive to the electrode potential for other compounds as pyridine and its derivatives whose enhancement is related to the SERS-CT mechanism given that the energy of the CT transition should be very dependent on the applied potential.^15^

In what follows, the analysis of the spectra will be based on the premise that metal and adsorbate form the metal–adsorbate (M–A) surface complex which is considered as a single chemical species. Although the composition and structure of such a complex is unknown, we have shown in previous works^8–19^ that this species and the effect of the electrode potential on it can be modeled by an adequate choice of the adsorbate bonded through the nitrogen atom of the pyridyl group to linear metallic Ag_*n*_^*q*^ clusters with different densities of charge. To be specific, we have selected the following linear silver clusters attached to *C*_2_(a)-BP4VA: Ag_3_^+^, Ag_5_^+^, Ag_7_^+^, Ag^0^_2_, Ag_7_^−^, Ag_5_^−^, Ag_3_^−^ (Fig. S3†). These clusters correspond to charge densities (*q*/*n*) ranging from +0.33 to −0.33 a.u., modelling Ag^0^_2_ the case of potential of zero charge. The M–A systems display a directional bond between the molecule and the metal through in which the nitrogen atom of the pyridyl fragment lies along the axis of the metal cluster (Fig. S3†); there are no other orientations or bonding interactions between adsorbate and metal, any attempt to find out other types of interactions was unfruitful, for example, bonding of the nitrogen atom with the metal at right angles or bonding of the molecule to the metal cluster through the anthracene skeleton.

**Fig. 5:**
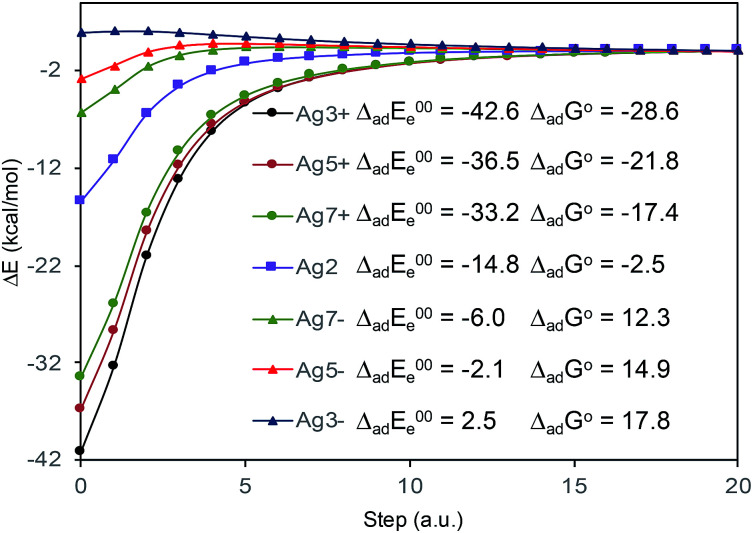
CAM-B3LYP/def2-TZVPP profiles of the potential energy surfaces for adsorption of BP4VA to the metal cluster. Δ_ad_*E*^00^_e_: adsorption electronic energy in kcal mol^−1^ corrected by zero-point energy. Δ_ad_*G*^o^: standard Gibbs energy of adsorption in kcal mol^−1^.

The absolute intensities of the SERS spectra depend on the *Γ* damping factor in inverse proportion. The effect of the damping factor is to decreases the emission of radiation of an excited system because it opens a channel for non-radiative deactivation.^26^ Thus, this factor is responsible for decreasing of the intensities of SERS bands, and it is as well responsible for attenuation or elimination of fluorescence in SERS. Although it is one of the worst understood magnitudes in the SERS phenomenon and in any spectroscopic technique, it is known that its effect increases as increases the interaction energy between the adsorbate and the metal surface.^26^ In this context, Fig. 5 shows the profiles of the potential energy surfaces obtained with the linear interpolation method for the selected M–A complexes. In this figure, it is clearly observed that the stability of the complex decreases as the charge of the metallic cluster is more negative, in accordance with the enhancement of the SERS as electrode potential becomes more negative.

With respect to the observed line shapes, the broadening of the bands can be attributed to (1) the contribution to the spectra of at least two conformers of BP4VA, (2) flat minima on the potential energy surfaces that arise from the torsional degrees of freedom of the vinylene and pyridyl groups as it is shown in Fig. 2, and (3) the bonding of the molecule the metal surface.

The explanation of the selective enhancement of the observed bands in the SERS spectra is the most interesting issue and the main objective of this work. To achieve this end, we have developed a methodology that consists of relating the electronic structure of the M–A complex (the transitions between electronic states) with its ability to predict the specific intensification of the Raman lines in resonant conditions. Briefly, the electronic structures are obtained from quantum chemistry calculations (time dependent functional theory in this work) and this information is applied to the equations of the vibronic approximation of the multi-state resonance Raman theory to estimate the spectra. Fig. 4b displays the calculated resonance Raman spectra of the silver-BP4VA complexes studied in this work for the most stable *C*_2_(a) conformer. *C*_s_ isomerization to *C*_2_(a) requires less than 1 kcal mol^−1^. On top of Fig. 4b (green) is represented what we call the pure charge transfer spectrum, that is, the spectrum that would arise from the hypothetic resonance between the neutral molecule (A) and its radical anion (A^−^) related to the ground and the CT excited state of the metal molecule complex. The calculated SERS spectra corresponding to the *C*_s_-conformer are given in Fig. S4,† and they do not differ noticeably with respect to the *C*_2_(a) results shown in Fig. 4b.

Any comparison between calculated and observed SERS spectra is not straightforward. There are two points that are important to highlight: (1) the charge distribution on the surface of the metal is not homogenous, in consequence, at each electrode potential, we can observe signals arising from metal clusters with different charges; (2) the potential of zero charge of a rough silver electrode is not known with certainty, at the very most, we can hypothesize that its range varies between −0.6 and −0.9 V, and therefore, the calculated spectrum of Ag_2_–BP4VA is correlated with the SERS recorded at −0.7 V in Fig. 4. However, in spite of these two drawbacks, the information that can be extracted from the comparison of the observed SERS spectra with the calculated ones is quite useful.

The calculations predict that the observed characteristic triad of bands in the 1550–1750 cm^−1^ region, which correspond to the calculated modes 8a[1] of anthracene (∼1640 cm^−1^), 8a of pyridine (∼1680 cm^−1^) and *ν*(C=C) of vinylene (1739 cm^−1^), respectively, are selectively enhanced in accordance with the experimental behavior. Likewise, the calculations predict correctly the intensity ratios of such modes, being the 8a[1] mode of anthracene the most intense band of the triad. The calculated spectra also show that the 8a[2] mode (∼1477 cm^−1^) of anthracene should be selectively intensified as the densities of charge of the complexes becomes more negative as it is observed when the electrode potential in the experiments becomes more negative. At this point, it is important to note that the calculated SERS spectra resemble the resonance Raman spectra of the isolated species. The active bands are almost the same in both kind of spectra, excepting the 8a mode of pyridine, which is active in the SERS spectra but missing in the RR of the isolated species. A fact that is in accordance with the pyridyl moiety interacting with the metal through the nitrogen atom, as it is proposed in this work. In contrast, the calculated SERS differ noticeably with respect to the calculated and observed normal Raman spectra (Fig. 1a).

**Fig. 6:**
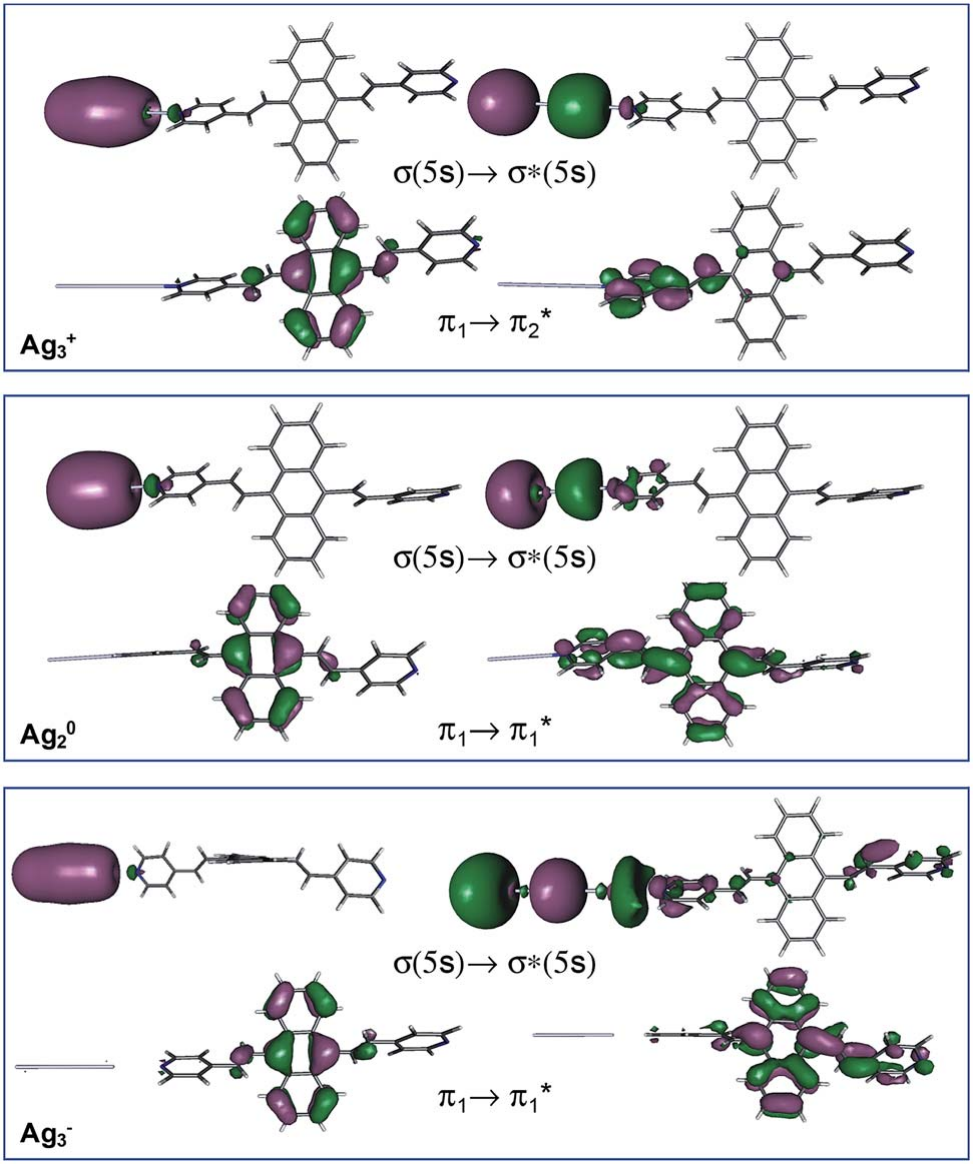
Representative molecular orbitals of the main electronic transitions of the M–A-complexes.

Table 1 summarizes the excitation energy and charge distribution of the more representative electronic states in the calculated SERS spectra of this work, that is, those states with higher contribution to the total intensity of each spectrum. In turn, Fig. 6 depicts the main orbitals of the relevant electronic states involved in the SERS spectra of three selected complexes. To be specific, the most significant electronic excitations correspond to (i) internal transitions of the metal [σ(5s) → σ*(5s)], what we call plasmon resonances, and (ii) internal transitions of the adsorbate (BP4VA) [π → π*] with strong character of intramolecular charge transfer. While the plasmon resonance is present in all of the spectra, the character of the π → π* transition depends on the charge of the complex. The π starting orbital is the π orbital of anthracene for all the complexes, however the π* orbital is localized on the vinylene fragment for positive complexes and on the anthracene for neutral and negative ones. In any case, the recorded SERS spectra are very similar and can be classified into the category of surface-enhanced resonance Raman scattering (SERRS). This assertion is reinforced by the SERS spectra registered at 785 nm (out of resonance) which are given in Fig. S5.† In these spectra, it is clearly observed that the mode 8a[1] of anthracene is not intensified with respect to modes 8a of pyridine and *ν*(C=C) of vinylene.

**Table 1:** Charge distribution of the excited states of the M–A complexes^a^^,^^b^

Species	State	Charges						*W* c	Assignment
		Δ*Q*_1_^d^	Δ*Q*_2_^d^	Δ*Q*_3_^d^	Δ*Q*_4_^d^	Δ*Q*_5_^d^	Δ*Q*_M_^d^		
Isolated	S_1_ (3.14)^e^	+0.05	−0.01	−0.01	−0.02	−0.02	*∅*	0.99	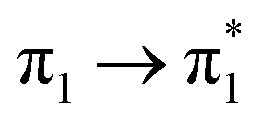
Anion	D_0_	−0.59	−0.07	−0.07	−0.13	−0.13	*∅*	1	(π*)^1^
Ag_3_^+^–BP4VA	S_2_ (2.00)	0	0	0	+0.02	0	−0.02	0.80	σ(5s) → σ*(5s)
	S_3_ (2.65)	+0.32	−0.09	+0.02	−0.22	+0.02	−0.05	0.60	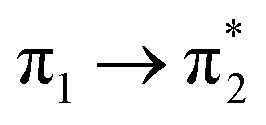
Ag_5_^+^–BP4VA	S_2_ (1.64)	0	0	0	0	0	0	0.80	σ(5s) → σ*(5s)
	S_4_ (2.74)	+0.31	−0.09	+0.02	−0.21	+0.02	−0.05	0.60	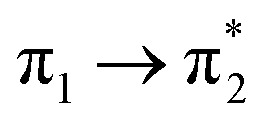
Ag_7_^+^–BP4VA	S_1_ (1.39)	0	0	0	0	0	0	0.83	σ(5s) → σ*(5s)
	S_6_ (2.79)	+0.29	−0.08	+0.01	−0.20	+0.02	−0.05	0.52	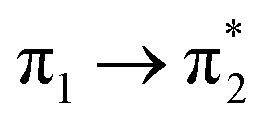
Ag^0^_2_–BP4VA	S_2_ (3.08)	+0.10	−0.03	−0.00	−0.06	−0.01	−0.01	0.98	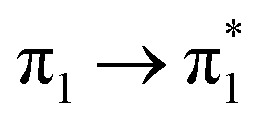
	S_3_ (3.32)	0	0	0	0	0	0	0.20	σ(5s) → σ*(5s)
Ag_7_^−^–BP4VA	S_3_ (1.69)	0	0	0	0	0	0	0.98	σ(5s) → σ*(5s)
	S_18_ (3.14)	0	0	0	0	0	0	0.04	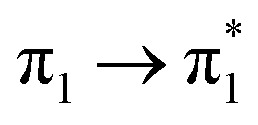
Ag_5_^−^–BP4VA	S_5_ (2.07)	0	0	0	0	0	0	0.99	σ(5s) → σ*(5s)
	S_16_ (3.14)							0.01	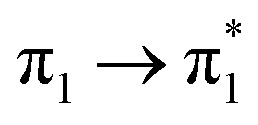
Ag_3_^−^–BP4VA	S_11_ (2.65)	0	0	0	0	0	0	0.98	σ(5s) → σ*(5s)
	S_15_ (3.14)	0	0	0	0	0	0	0.03	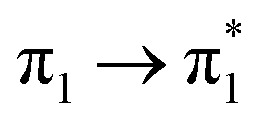

a CAM-B3LYP/def2TZVPP.

b Negative transferred charge (Δ*Q*_j_) implies that the fragment “j” increases its negative charge.

c Weight of the electronic state in the calculated spectrum.

d Transferred charge on 1: anthracene; 2,3: vinyl; 4,5: pyridyl; M: silver cluster.

e In parenthesis, excitation energy in eV.

At this point, it must be noted that in all the studied complexes in this work, there exist charge transfer (CT) states above and below the strongly allowed states which dominate the SERS spectra (Table S2†). Such CT states do not contribute to the activation of any SERS band due to they have very small or zero oscillator strengths. In other words, these CT states are not accessible by irradiation of the sample, but they can be populated by a non-radiative mechanism. In this context, it is interesting to stress that the calculated SERS for the most negative complex (Ag_3_^−^–BP4VA) matches with the calculated intensities for the isolated species involving the neutral and the respective anion (top Fig. 4b and S6†), however this coincidence is only accidental, given that there are not significant charge transfer contributions to the calculated SERS of Ag_3_^−^–BP4VA. This fact is rather surprising, because the similitudes between both kinds of spectra could be taken as a prove of the participation of the charge transfer states in the SERS of pyridine derivatives.^16,21,22^

To finish, due to the adsorbate is a bidentate system, we have studied the SERS of BP4VA bonded to two symmetric silver clusters trough both pyridyl moieties: Ag_2_–BP4VA–Ag_2_ (Fig. S7†). The spectrum of the bidentate complex is identical to the Ag_2_–*C*_2_(a)-BP4VA or Ag_2_–*C*_s_-BP4VA results. Therefore, we cannot distinguish between single or double union of the adsorbate to the metal. However, we can anticipate that the bidentate union is less probable because it requires a very specific structure of the local active sites of the metal.

## Conclusions

We have studied the SERS spectra of 9,10-bis((*E*)-2-(pyridin-4-yl)vinyl) anthracene (BP4VA, Scheme 1) in an electrochemical cell on silver working electrode at different potentials. The spectra have been interpreted on the basis of resonance Raman vibronic theory assisted by DFT calculations. The experimental behavior has been explained on the basis of the electronic structure of silver complexes where the adsorbate–metal interaction occurs through the nitrogen atom of the pyridyl moiety. The enhancement factor of the electrode from the chemical enhancement and the electromagnetic enhancement mechanisms amounts to 2.25 (Table S3†).

Concerning the electron donor–acceptor properties of the adsorbate, it is shown that the charge-transfer excited states of BP4VA do not play a relevant role in SERS given that they are not optically active. It is an internal transition to an excited state of the adsorbate, which is localized in the anthracene framework, that dominates the SERS spectra. Afterwards, charge transfer states would be populated by means of non-radiative processes (internal conversion or intersystem crossing) in a short time scale,^38,39^ that is, by an ultrafast process. In other words, irradiation of BP4VA interacting with an appropriate surface creates an effective charge separation. Thus, this finding is relevant, for example, in the photovoltaic field^40–42^ because it shows the ability of SERS spectroscopy in conjunction with quantum chemical calculations to characterize the electronic properties of a material. In addition, it has the advantage to require a very small amount of product.

## Methods

### Experimental section

BP4VA was synthesized by one of us (E. I.) as described in a recent article.^43^ SERS spectra have been recorded under 514.5 nm excitation by using a Renishaw Invia micro-Raman spectrometer. The microscope was equipped with a macro objective (*f*: 30 mm). Wire 2.0 from Renishaw has been used for spectral data acquisition and manipulation. The spectral resolution was set at 2 cm^−1^. The electrochemical instrument used to record the SERS spectra consists of a CH potentiostat (model 600E) and a three electrodes cell with (i) a platinum counter electrode, (ii) a Ag/AgCl/KCl sat. reference electrode to which all the electrode potentials were referred to, and (iii) a pure silver working electrode were used. The working electrode was electrochemically activated in order to produce the required SERS active nanostructures by maintaining the electrode potential at −0.5 V and then subjecting it to seven pulses at +0.6 V for 2 seconds. A 0.1 M aqueous solution of Na_2_SO_4_ has been employed as electrolyte in the activation procedure. Previously, the silver working electrode has been polished with 0.30 and 0.05 μm alumina (Büehler). SERS spectra of BP4VA/Na_2_SO_4_ with a concentration of 10^−4^ M/0.1 M were recorded. Pure water obtained from a Milli-Q instrument was employed in the solutions.

The series of spectra, from 0.0 V up to −1.2 V, were recorded in triplicate being each spectrum recorded with the accumulation of 1 scan and a 10 s exposure time. The laser power at the sample was 6.0 mW. Spectra were not corrected for instrument response. There were no visible or spectroscopic signs of sample damage that were evident after data acquisition and also there were no significant fluctuations for a particular spectrum in all the sequence of spectra. The relative intensities of the bands for a particular spectrum at a specific electrode potential keep almost constant in the three series of spectra recorded, indicating a good reproducibility of the data.

### Computational details

SERS and resonance Raman spectra have been calculated in accordance with the vibronic theory of Albrecht^34–36^ modified by a multi-state version^44,45^ which considers only Franck–Condon factors. It is assumed the small displacement approximation and the independent mode displaced harmonic oscillator (IMDHO) model. It is assumed as well that there is no mode rotation in the states under consideration.

The integrals of vibrational overlapping, Δ_*k*,e_, are obtained using the recurrence formulae of Manneback^46^ with the gradient approximation, eqn (1).1
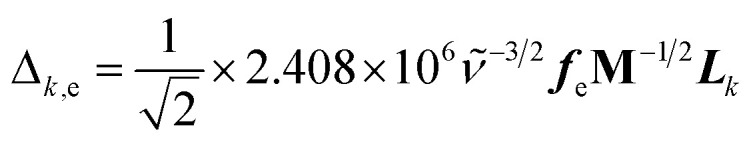
where ***f***_e_ is the force row vector of electronic state e, **M** is the 3*N* × 3*N* diagonal matrix of atomic masses, and ***L***_*k*_ is the column eigenvector of the Hessian matrix for mode *k*-th.

To be specific, we have implemented a modified expression of the intensity equation given by Long, eqn (2)2
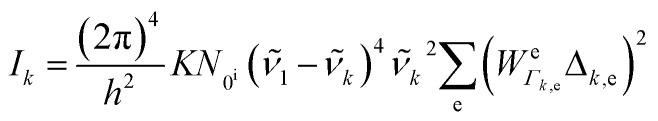
where *K* is a constant for a given experimental condition and a given irradiance of the incident radiation of wavenumber *ν̃*_1_; *N*_0^i^_ is the Boltzmann population of the vibrational ground state; *ν̃*_*k*_ is the wavenumber of *k*-th mode; 
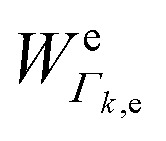
 is given by eqn (3) and depends on the transition dipole moment *μ*^0^_e_; *ν̃*_e_, the energy difference between the excited electronic state e and the ground state; the wavenumber *ν̃*_*k*_ of *k* mode and; the damping factor *Γ*_*k*,e_ which is related to the lifetime of the transition and is taken as 0.2*ν̃*_*k*_ in this work.3
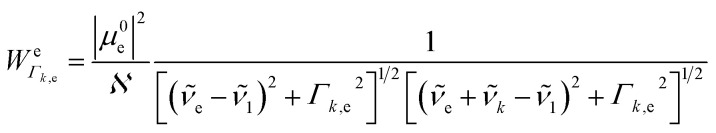

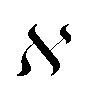
 is the normalization constant given by eqn (4).4
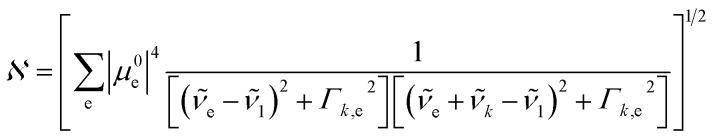


All of the quantum chemical calculations have been performed with the density functional theory by applying the hybrid exchange–correlation functional CAM-B3LYP,^47^ in conjunction with the def2-TZVPP basis sets for the first and second row elements and the corresponding electron core potential for the silver atoms.^48,49^ The metal–molecule complexes have been optimized in internal coordinates. The only constrain is that the geometries of the metallic clusters are kept frozen at the linear geometry calculated for the corresponding isolated systems. Each stationary point was characterized by its analytically computed harmonic frequencies. Mulliken's atomic charges have been used. The electronic structure calculations were carried out with the package of programs GAUSSIAN16.^50^ Adsorption energies were determined by using the standard expressions of statistical hermodynamics with programs implemented by us.^51,52^ Molecular structures, vibrational wavenumbers and molecular orbitals were analysed with the help of MacMolPlt,^27^ MOLDEN^53^ and GABEDIT^54^ programs.

## Conflicts of interest

There are no conflicts to declare.

## Supplementary Material

RA-009-C9RA01269A-s001
