# Supplementary material for: Application of surface-enhanced resonance Raman scattering (SERS) to the study of organic functional materials: electronic structure and charge transfer properties of 9,10-bis((E)-2-(pyridin-4-yl)vinyl)anthracene
Source: RSC Adv. 2019 May 9;9(25):14511–9. doi: 10.1039/c9ra01269a (PMC9064130; doi:10.1039/c9ra01269a)
Supplement: RA-009-C9RA01269A-s001 [file RA-009-C9RA01269A-s001.pdf]

Electronic Supplementary Material (ESI) for RSC Advances.
This journal is © The Royal Society of Chemistry 2019

RSC

ADVANCES

Electronic Supplementary Information

Application of surface-enhanced resonance Raman scattering (SERS) to the study of organic functional materials: electronic structure and charge transfer properties of 9,10-bis((*E*)-2-(pyridin-4-yl)vinyl)anthracene

by

Juan Soto,* Elizabeth Imbarack, Isabel López-Tocón, Santiago Sánchez-Cortés, Juan C. Otero , and Patricio Leyton

CONTENTS

Fig. S1 Calculated normal Raman spectrum of C_s-BP4VA at 514.5 and 1064 nm...S3

Fig. S2 Graphical representation of normal modes of C_s-BP4VA...................S4

Table S1 Assignment of Raman active modes of C_s-BP4VA. S6

Fig. S3 CAM-B3LYP/def2-TZVPP geometries of the MA complexes: (i) Ag_3^+; (ii) Ag_5^+; (iii) Ag_7^+; (iv) Ag_2^0; (v) Ag_7^-; (vi) Ag_5^-; (vii) Ag_3^-.. ....S7

Fig S4 Calculated SERS spectra of (C_s-BP4VA)-silver complexes at λ_{exc} = 514.5 nm. Spectra convoluted with a Voigt function (1:1) of HWHM = 5 cm^{-1}. (CAM-B3LYP/def2-TZVPP). On top (green) S_0-D_0 charge transfer spectrum of the isolated molecule. S8

Table S2 Charge transfer states of the BP4VA-metal complexes. S9

Fig S5 SERS spectra at λ_{exc} = 785 nm of BP4VA/Na_2SO_4 (10^{-4} M/0.1 M) aqueous solution on silver at different electrode potentials (reference electrode Ag/AgCl/KCl sat.). S10

Fig S6 (a) CT spectra of the hypothetic S_0-D_0 transition for BP4VA conformers. CAM-B3LYP/def2-TZVPP. (i) C_2-BP4VA; (ii) C_i-BP4VA; (iii) C_s-BP4VA; (iv) C_2-BP4VA (b) Electron acceptor orbital of the radical anion. S11

Fig S7 Calculated SERS spectra of BP4VA-silver complexes at λ_{exc} = 514.5 nm: (a) C_2(a)-Ag_2; (b) Ag_2-C_2(a)-Ag_2; (c) C_s-Ag_2. Spectra convoluted with a Voigt function (1:1) of HWHM = 5 cm^{-1}. (CAM-B3LYP/def2-TZVPP). .S12

Table S3 Relative intensities of the resonant SERS band recorded at 1555 cm^{-1} (514.5nm) and 1558 (785nm) with respect to that recorded at 1627 and 1631 cm^{-1}..S13

The Raman spectra of C_s-BP4VA given in Figs. 1 and S1 have been calculated with Eq. (S1), where the intensity of *k*-th mode is given by the differential cross section (units of cm^2/sr)

$$I_k = \frac{d\sigma}{d\Omega} = \frac{\pi^2}{\varepsilon_0^2}(\tilde{\nu}_1 - \tilde{\nu}_k)^4 \frac{h}{8\pi^2 c\tilde{\nu}_k}(S_k/45)\frac{1}{1 - exp(hc\tilde{\nu}_k/k_B T)} \quad \text{(S1)}$$

S_k is the scattering factor (units in Å^4/amu) calculated with the polarizability gradient method

$$S_k = 45\left(\frac{d\alpha}{dQ_k}\right)^2 + 7\left(\frac{d\gamma}{dQ_k}\right)^2 \quad \text{(S2)}$$

ε_0 is the permittivity of vacuum, c is the speed of light, h is the Planck constant, k_B is the Boltzmann constant, T is the temperature, $\tilde{\nu}_1$ is the wavenumber of the incident light, $\tilde{\nu}_k$ is the wavenumber of the *k*-th vibrational mode.

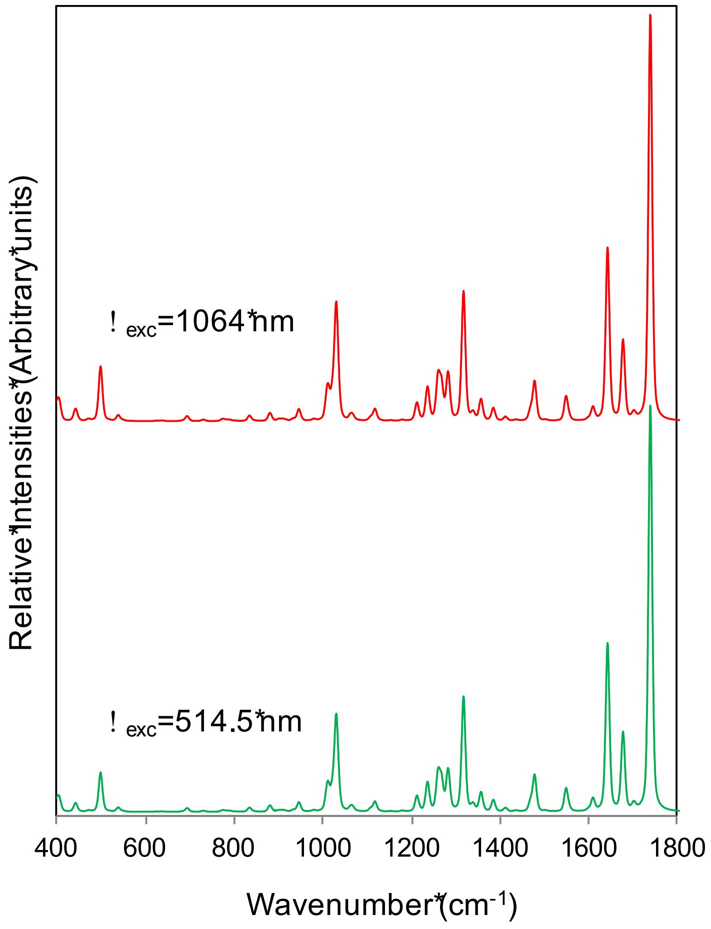

Fig S1. Calculated normal Raman spectrum of C_s-BP4VA at 514.5 and 1064 nm. Convoluted with a Voigt function (1:1) and HWHM = 5 cm^{-1}.

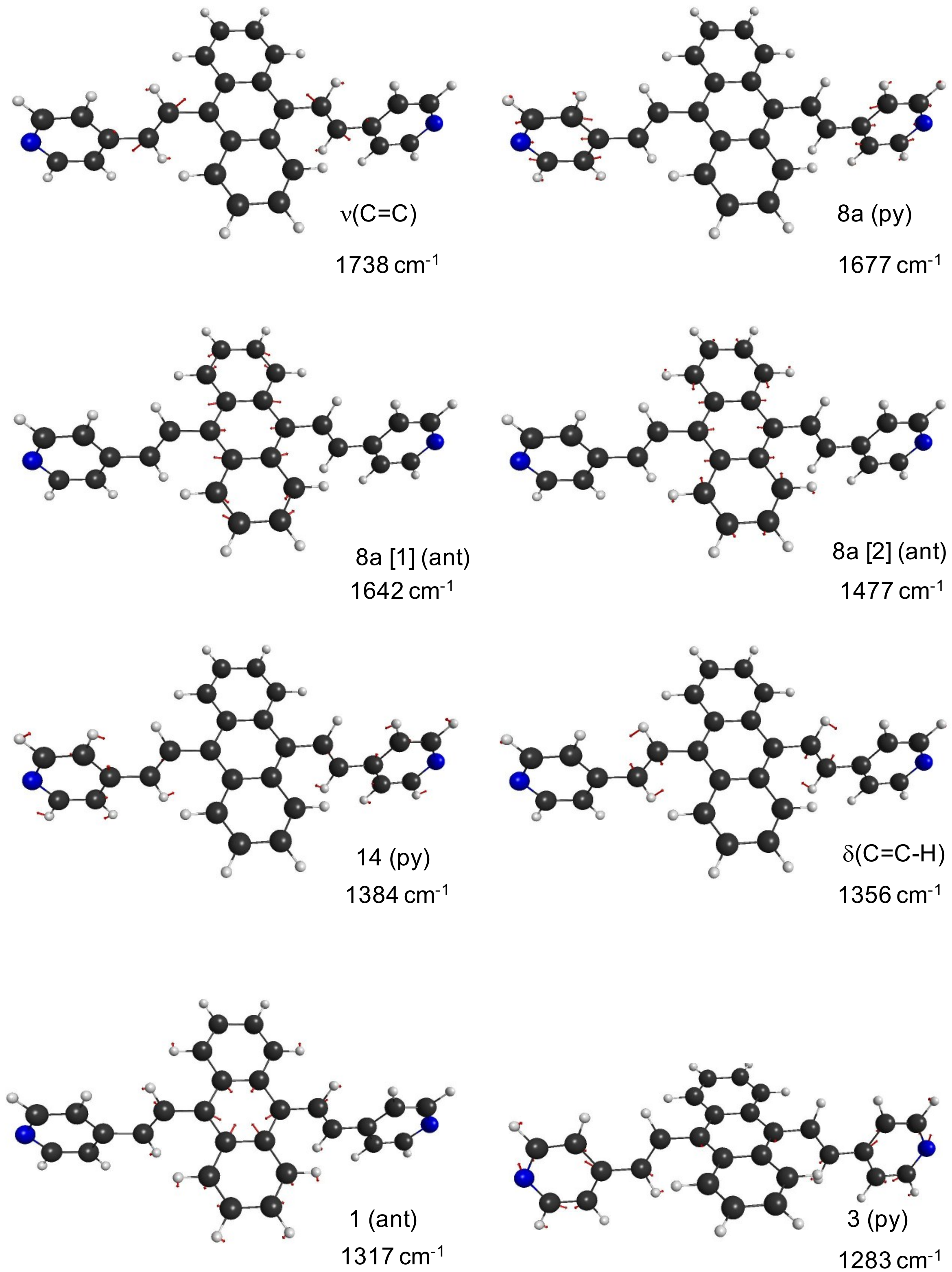

Fig S2 (continue). Active normal Raman modes of C_s-BP4VA.

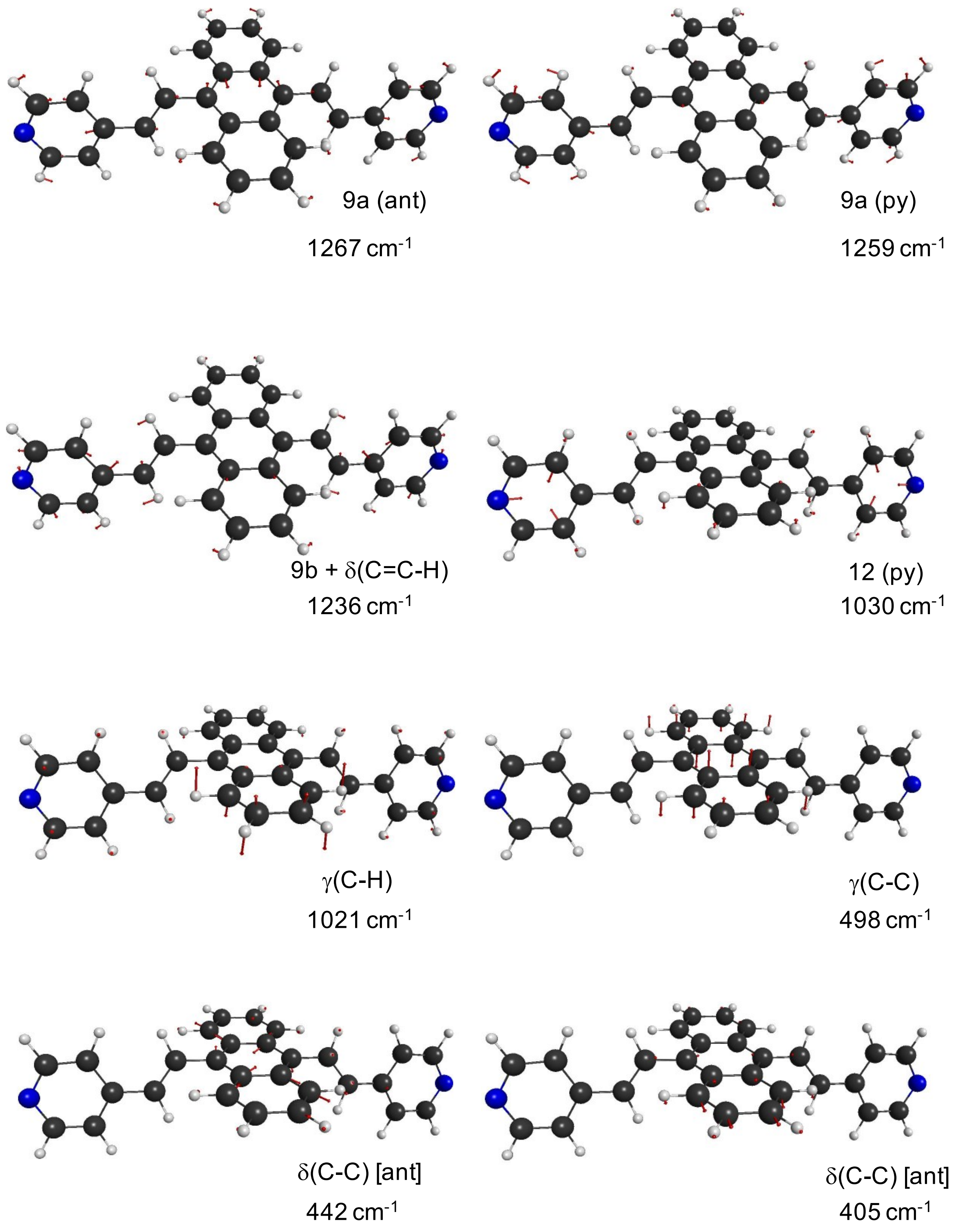

Fig S2 (continuation). Active normal Raman modes of C_s-BP4VA.

Table S1. Vibrational assignment of the Raman spectrum of solid BP4VA.

Observed[a]	Calculated[b]	Ratio	Assignment
1636	1738	0.94	ν(C=C) vinylene
1596	1677	0.95	8(a) pyridine
1559	1642	0.95	8a [1] anthracene
1412	1477	0.96	8a [2] anthracene
1339	1384	0.97	14 pyridine
1308	1356	0.96	δ(C=C-H) hydrogen bending
1268	1317	0.96	1 anthracene
1237	1283	0.96	3 pyridine
1220	1267	0.96	9a anthracene
1200	1259	0.95	9a pyridine
1175	1235	0.95	9b + δ(C=C-H)
1083	1117	0.97	
1027	1065	0.96	
992	1030	0.96	12 pyridine
971	1021	0.95	γ(C-H)
477	498	0.96	γ(C-C)
431	442	0.98	δ(C-C)
398	405	0.98	δ(C-C)

[a]Observed frequencies in cm^{-1} (Fig. 1). [b]CAM-B3LYP/def2TZVPP C_s-conformer.

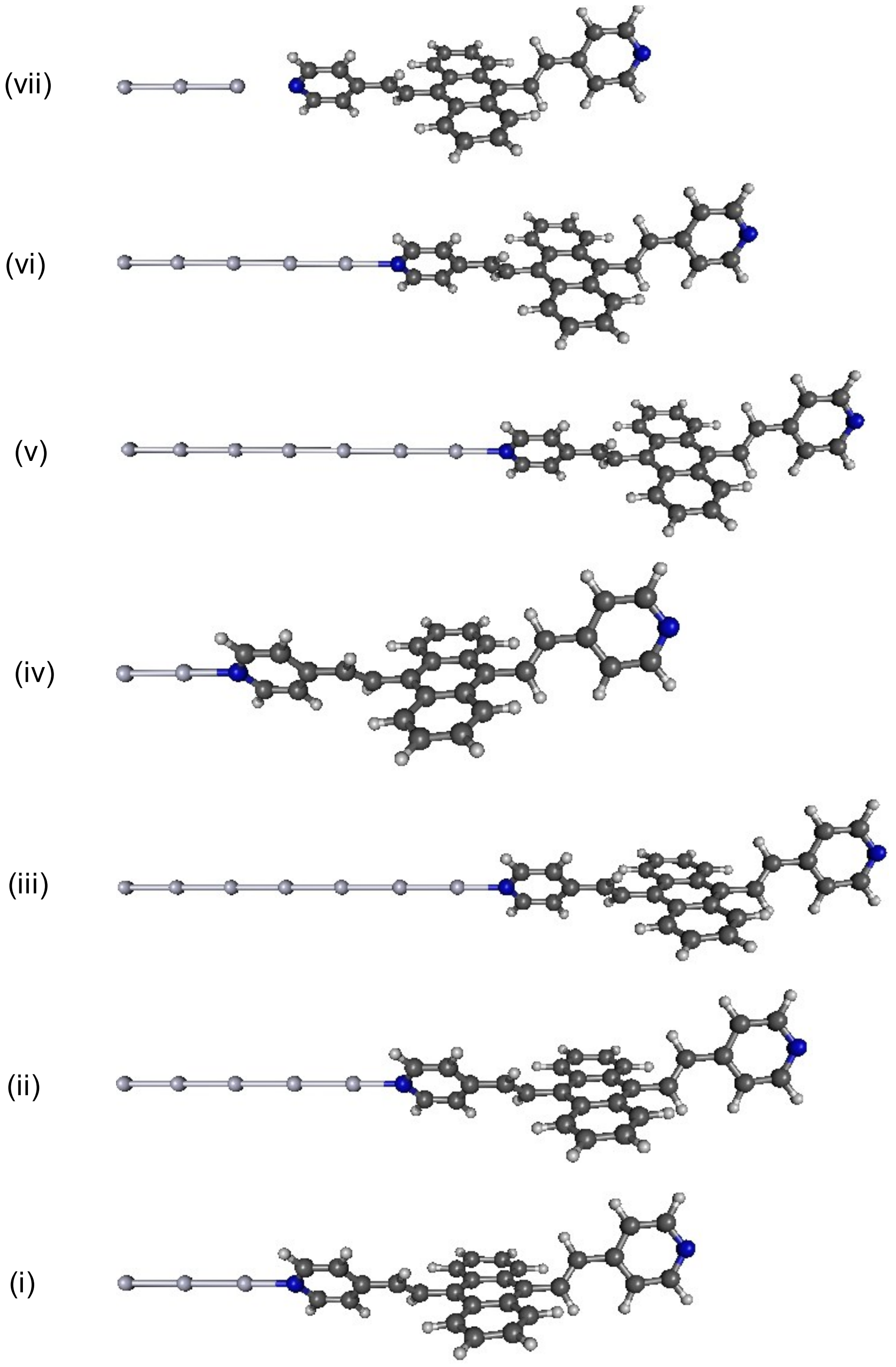

Fig. S3. CAM-B3LYP/def2-TZVPP geometries of the MA complexes: (i) Ag_3^+; (ii) Ag_5^+; (iii) Ag_7^+; (iv) Ag_2^0; (v) Ag_7^-; (vi) Ag_5^-; (vii) Ag_3^-.

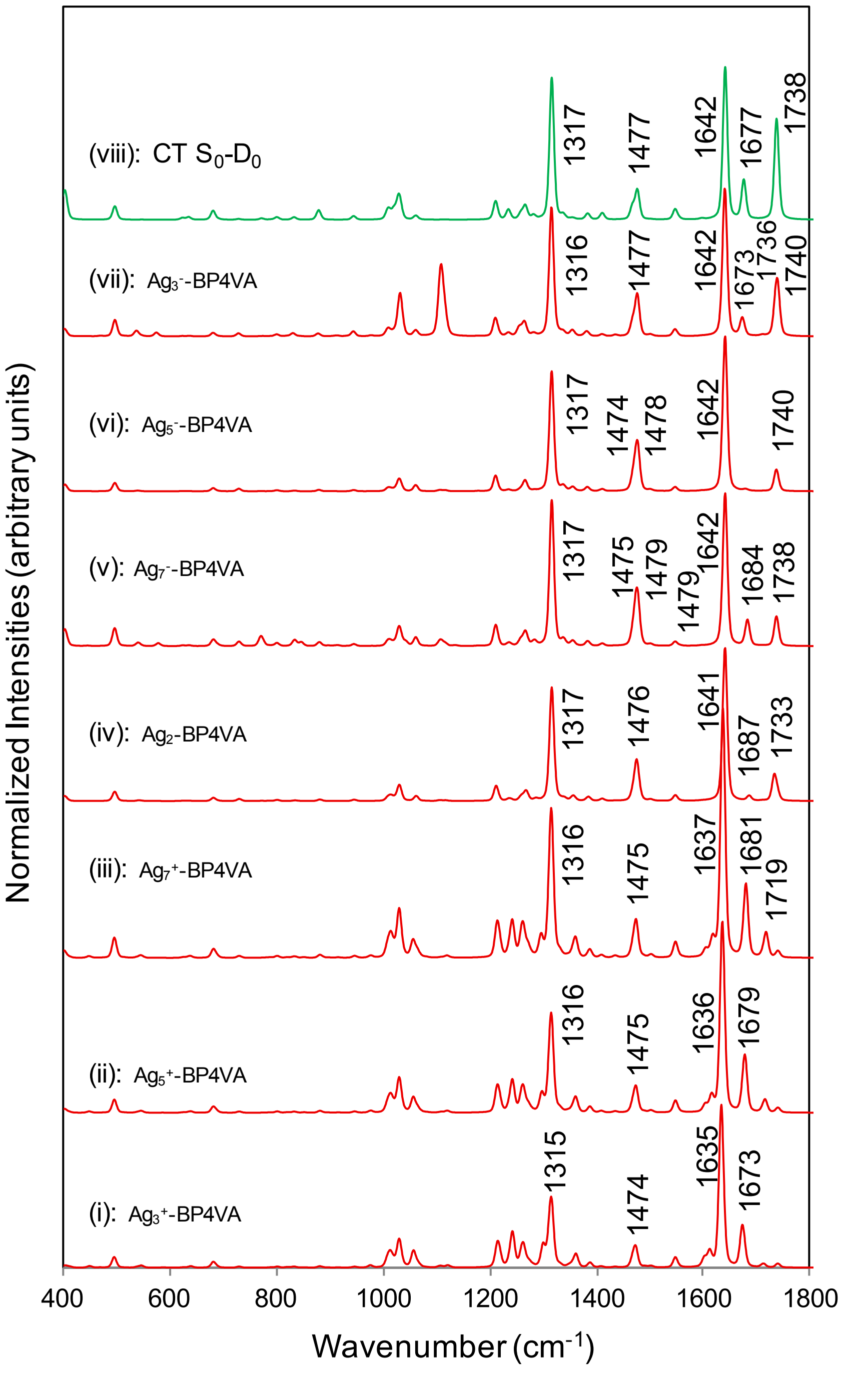

Fig S4. Calculated SERS spectra of (C_s-BP4VA)-silver complexes at λ_{exc} = 514.5 nm. Spectra convoluted with a Voigt function (1:1) of HWHM = 5 cm^{-1}. (CAM-B3LYP/def2-TZVPP). On top (green) S_0-D_0 charge transfer spectrum of the isolated molecule.

Table S2. Charge transfer states of the BP4VA-metal complexes.[a,b]

**Species**	**State**[c]	ΔQ_M[d]	ΔQ_1[d]	ΔQ_2[d]	ΔQ_3[d]	ΔQ_4[d]	ΔQ_5[d]
Ag_3^+-BP4VA	S_1 (1.33)	-0.82	+0.61	+0.04	+0.05	+0.03	+007
	S_4 (2.88)	-0.82	+0.67	+0.00	+0.04	+0.02	+0.10
Ag_5^+-BP4VA	S_1 (1.42)	-0.85	+0.63	+0.05	+0.06	+0.04	+0.08
	S_5 (2.96)	-0.86	+0.69	+0.01	+0.04	+0.03	+0.10
Ag_7^+-BP4VA	S_2 (1.54)	-0.87	+0.64	+0.05	+0.6	+0.05	+0.08
	S_7 (2.91)	-0.87	+0.63	+0.05	+0.06	+0.05	+0.08
Ag_2^0-BP4VA	S_1(2.69)	+0.81	-0.18	-0.13	-0.02	-0.44	-0.04
	S_5 (3.83)	+0.80	-0.05	-0.02	-0.01	-0.71	-0.02
Ag_7^--BP4VA	S_2 (1.63)	+0.86	-0.27	-0.12	-0.04	-0.35	-0.09
	S_4(2.02)	+0.90	-0.47	-0.10	-0.05	-0.19	-0.10
	S_5(2.13)	+0.93	-0.25	-0.02	-0.20	-0.03	-0.44
	S_6(2.51)	+0.85	-0.08	-0.03	-0.01	-0.70	-0.03
	S_7(2.53)	+0.85	-0.35	-0.09	-0.04	-0.28	-0.08
Ag_5^--BP4VA	S_2 (1.27)	+0.88	-0.23	-0.14	-0.04	-0.39	-0.09
	S_3 (1.68)	+0.93	-0.26	-0.02	-0.19	-0.04	-0.43
	S_4 (1.68)	+0.91	-0.50	-0.08	-0.06	-0.16	-0.11
	S_6 (2.17)	+0.86	-0.06	-0.03	-0.01	-0.74	-0.02
	S_7 (2.31)	+0.93	-0.76	-0.02	-0.02	-0.06	-0.06
	S_8 (2.35)	+0.92	-0.48	-0.08	-0.06	-0.18	-0.12
	S_9 (2.54)	+0.89	-0.28	-0.14	-0.04	-0.35	-0.09
	S_{10}(2.71)	+0.94	-0.07	-0.01	-0.03	-0.03	-0.80
Ag_3^--BP4VA	S_4 (1.53)	+0.96	-0.55	-0.07	-0.07	-0.14	-0.13
	S_5 (1.78)	+0.95	-0.75	-0.02	-0.02	-0.10	-0.05
	S_6 (1.87)	+0.91	-0.07	-0.02	-0.01	-0.78	-0.02
	S_7 (2.12)	+0.96	-0.07	-0.01	-0.03	-0.05	-0.80
	S_8 (2.42)	+0.94	-0.24	-0.13	-0.07	-0.33	-0.17
	S_9 (2.47)	+0.95	-0.64	-0.02	-0.03	-0.12	-0.13
	S_{10}(2.54)	+0.95	-0.33	-0.07	-0.12	-0.18	-0.26
	S_{14}(3.04)	+0.93	-0.37	-0.10	-0.06	-0.22	-0.17

[a]CAM-B3LYP/def2-TZVPP. [b]Negative transferred charge implies that the fragment increases its electron charge. [c]Excitation energy in eV. [d]Transferred charge on M: silver cluster; 1: anthracene; 2,3: vinyl; 4,5: pyridyl.

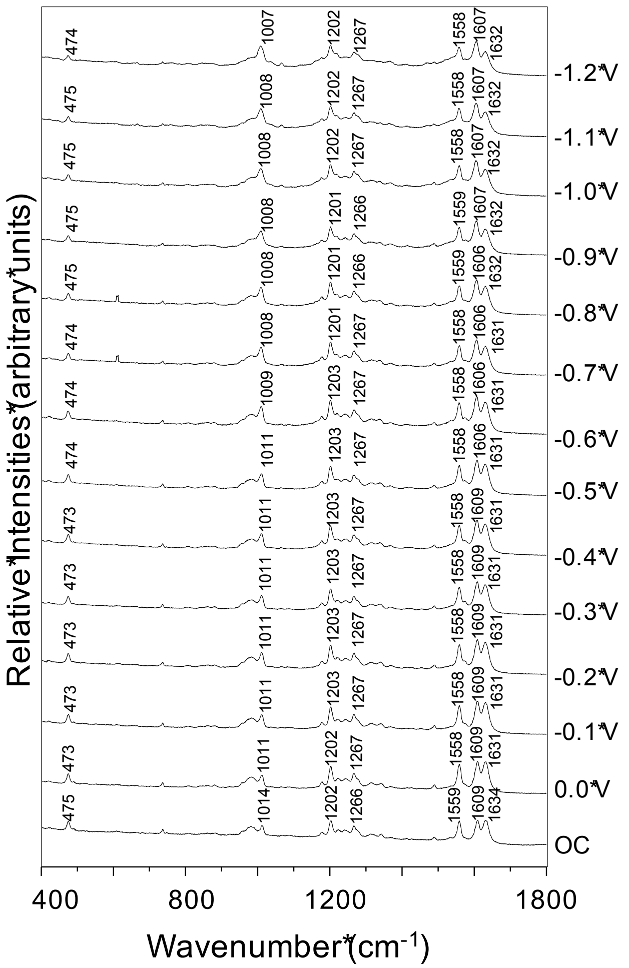

Fig S5. SERS spectra at λ_{exc} = 785 nm of BP4VA/Na_2SO_4 (10^{-4} M/0.1 M) aqueous solution on silver at different electrode potentials (reference electrode Ag/AgCl/KCl sat.).

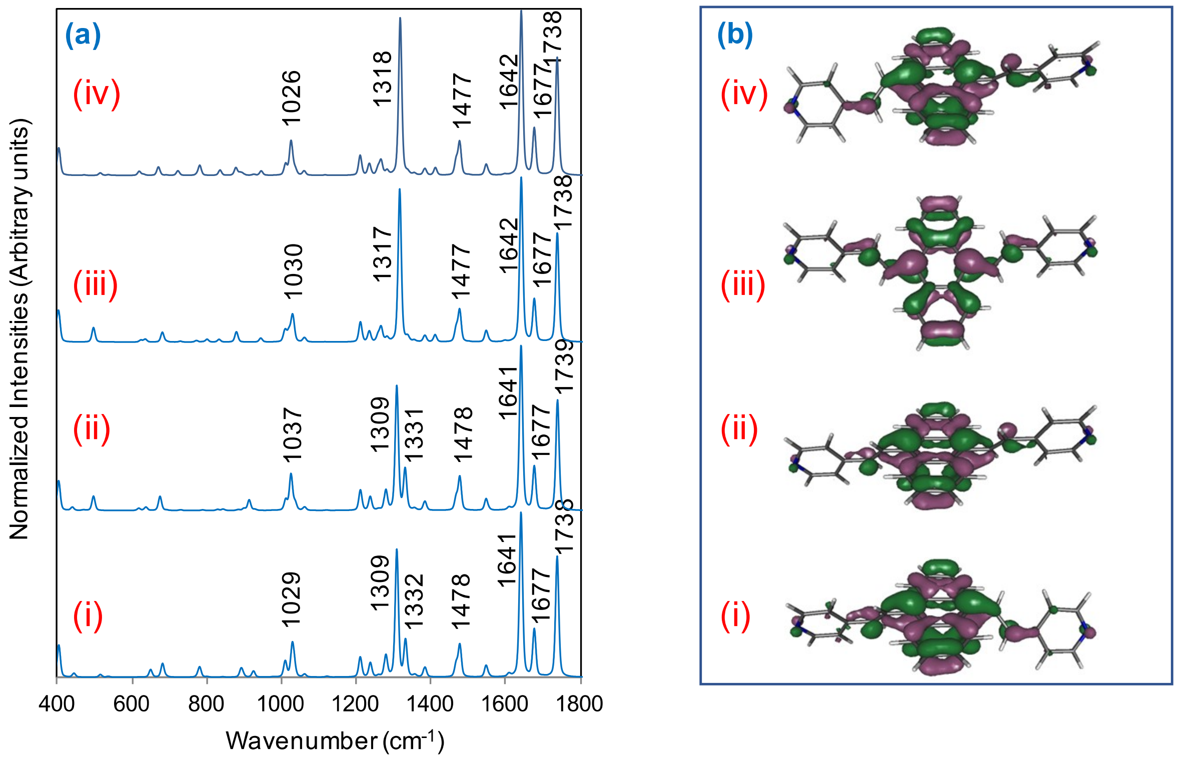

Fig S6. (a) CT spectra of the hypothetic S_0-D_0 transition for BP4VA conformers. CAM-B3LYP/def2-TZVPP. (i) C_2-BP4VA; (ii) C_i-BP4VA; (iii) C_s-BP4VA; (iv) C_2-BP4VA. (b) Electron acceptor orbital of the radical anion.

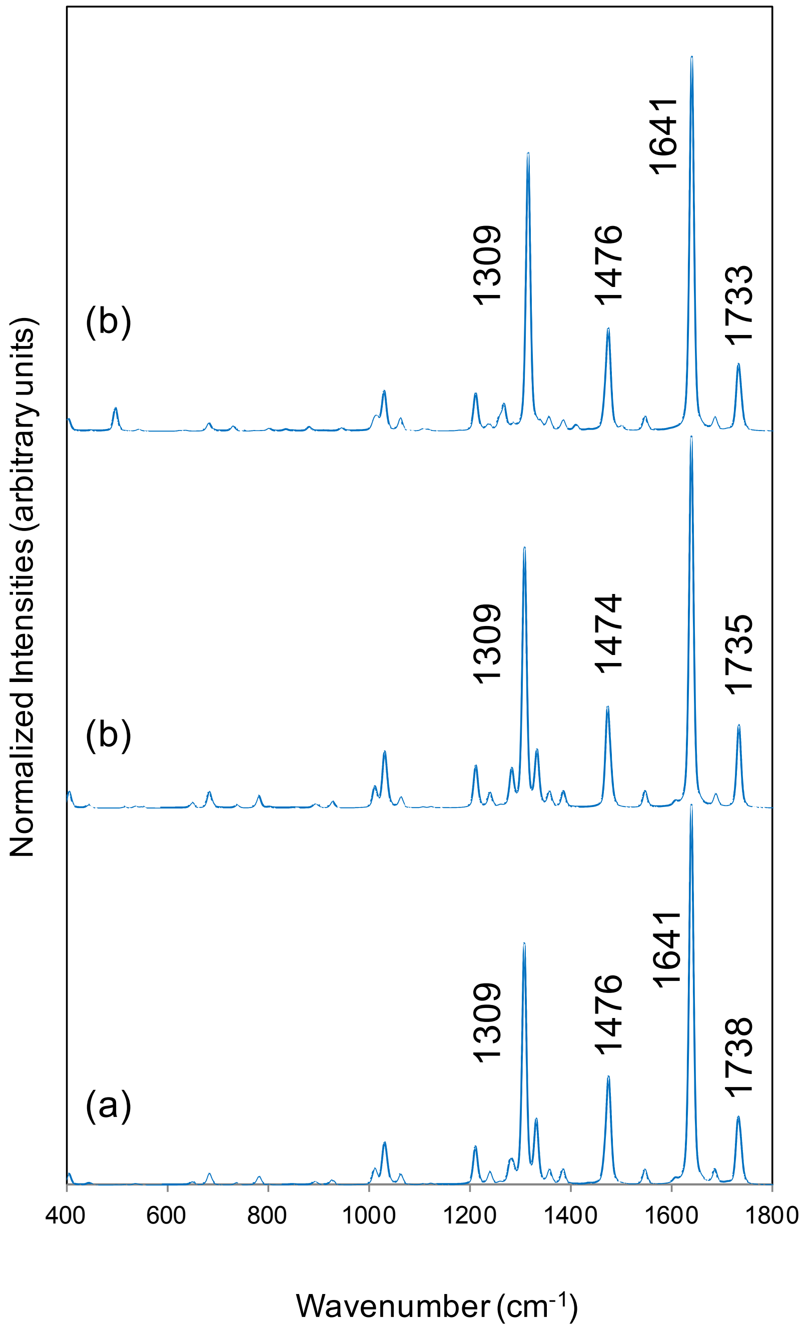

Fig S7. Calculated SERS spectra of BP4VA-silver complexes at λ_{exc} = 514.5 nm: (a) C_2(a)-Ag_2; (b) Ag_2-C_2(a)-Ag_2; (c) C_s-Ag_2. Spectra convoluted with a Voigt function (1:1) of HWHM = 5 cm^{-1}. (CAM-B3LYP/def2-TZVPP).

Table S3. Relative intensities of the resonant SERS band recorded at 1555 cm^{-1} (514.5nm) and 1558 (785nm) with respect to that recorded at 1627 and 1631 cm^{-1}.

**Electrode Potential /V**	**Relative Intensity**		**Resonance factor**
	514.5 nm I_{1555}/I_{1627}	785 nm I_{1558}/I_{1631}	514.5nm/785nm
OC	2.22	0.92	2.41
0.0	2.12	0.89	2.38
-0.1	2.10	0.88	2.38
-0.2	2.09	0.93	2.24
-0.3	2.21	0.93	2.38
-0.4	2.30	0.93	2.47
-0.5	2.10	0.94	2.23
-0.6	2.12	0.99	2.14
-0.7	2.21	0.99	2.23
-0.8	2.24	0.99	2.26
-0.9	2.20	1.08	2.04
-1.0	2.25	1.16	1.93
-1.1	2.26	1.00	2.26
-1.2	2.26	1.27	1.78
		**Statistics Data**[a]	
		Mean	2.25
		Standard Deviation	0.15

[a]Obtained considering all data except the last value obtained at -1.2 V.
